# A Critical Analysis of Quadratic Boost Based High-Gain Converters for Electric Vehicle Applications: A Review

**DOI:** 10.3390/s24072186

**Published:** 2024-03-28

**Authors:** Madhav Kumar, Kaibalya Prasad Panda, Ramasamy T. Naayagi, Ritula Thakur, Gayadhar Panda

**Affiliations:** 1 Department of Electrical Engineering, National Institute of Technology Meghalaya, Shillong 793003, India; madhavkumar523@gmail.com (M.K.); gayadhar.panda@nitm.ac.in (G.P.); 2 Department of Electrical Engineering, School of Energy Technology, Pandit Deendayal Energy University, Gandhinagar 382007, India; kaibalyapanda.nit@gmail.com; 3 School of Electrical and Electronic Engineering, Newcastle University in Singapore, Singapore 567739, Singapore; 4 Department of Electrical Engineering, National Institute of Technical Teachers Training & Research, Sector 26, Chandigarh 160019, India; ritula.thakur@gmail.com

**Keywords:** electric vehicle (EV), boost converter, quadratic boost converter (QBC), high-gain converter, boosting techniques

## Abstract

Conventional DC-DC boost converters have played a vital role in electric vehicle (EVs) powertrains by enabling the necessary voltage to increase to meet the needs of electric motors. However, recent developments in high-gain converters have introduced new possibilities with enhanced voltage amplification capabilities and efficiency. This study discusses and evaluates the state-of-the-art high-gain DC-DC converters for EV applications based on the Quadratic Boost Converter (QBC). Research into innovative topologies has increased in response to the increasing demand for efficient and high-performance power electronic converters in the rapidly expanding EV industry. Due to its ability to provide more significant voltage gains than conventional boost converters, the QBC has become a viable option for meeting the unique requirements of EV power systems. This survey focuses on the efficiency, power density, and overall performance parameters of QBC-based high-gain converters. The literature review provides a foundation for comprehending power electronics converters’ trends, challenges, and opportunities. The acquired knowledge can enhance the design and optimization of high-gain converters based on the QBC, thereby fostering more sustainable and efficient power systems for the expanding electric mobility industry. In the future, the report suggests that investigating new high-gain converter design methodologies will reduce component stress and enhance the intact system efficiency.

## 1. Introduction

Promoting an electric vehicle (EV)-based transportation system in India is being undertaken by the government, industry, and academia to mitigate daily transportation emissions and minimize environmental hazards. EVs use renewable energy sources or the electric grid for charging, accounting for approximately 25–30% of India’s total greenhouse gas emissions [1]. Governments across the globe are enacting subsidies and legislation to foster the adoption of EVs, as they are recognized for their potential to yield various societal advantages, including heightened safety standards, enhanced public health outcomes, a robust domestic economy, and a more environmentally sustainable future. Fossil fuels present substantial hazards to the Earth’s ecosystem, prompting numerous countries to shift towards renewable energy sources to achieve environmental sustainability and economic feasibility [2].

**Figure 1:**
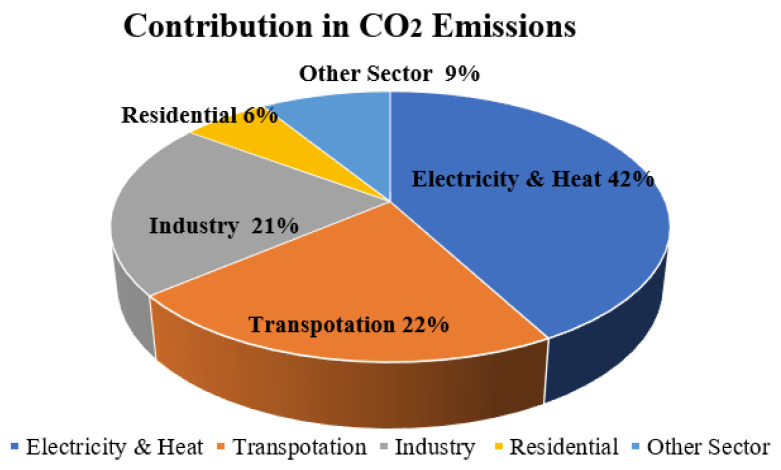
The percentage contribution of CO_2_ emissions by different sectors.

**Figure 2:**
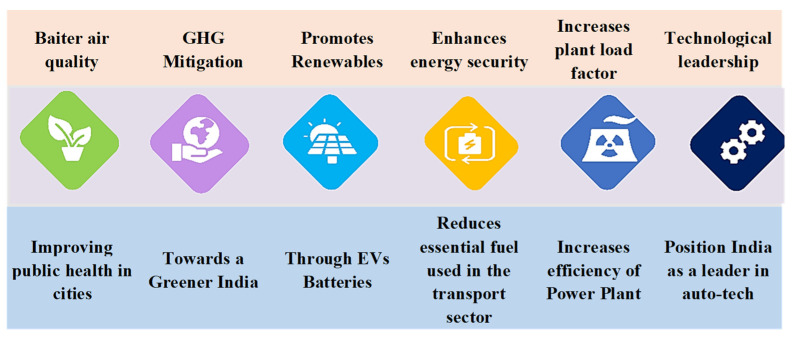
Social, environmental, and economical aspects of electric vehicles.

Human survival depends on maintaining a habitable environment, and fossil fuels have a finite supply. EVs are being promoted as a viable transportation option because of their efficiency and lack of environmental impact. As more people learn about the advantages of EVs, that number is projected to rise to over 100 million by 2030. As Asian nations work to increase EV uptake, decrease CO_2_ emission reported in Figure 1, and entice investors, the region’s EV sector stands to flourish. There will be 27,81,69,631 conventional cars and 13,34,381 EVs on Indian roads by 2022. The e-Vahan portal is managed by the Ministry of Road Transport and Highways and contains detailed information on EV sales in India and worldwide [3,4]. The social, environmental, and economic potential gains from EVs are shown in Figure 2.

**Figure 3:**
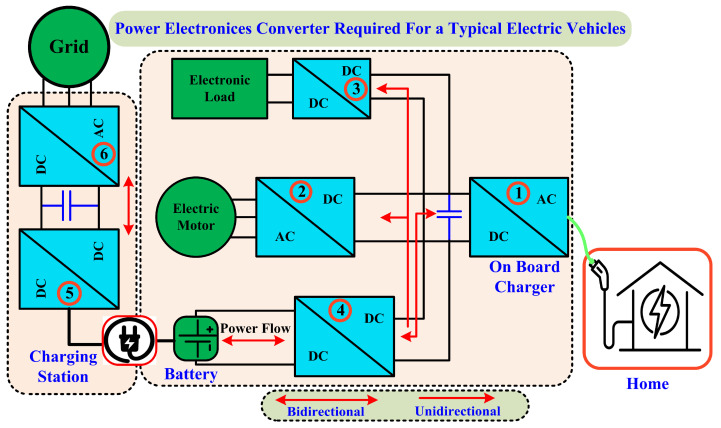
Importance of power electronics converter in EV system.

EVs’ social, environmental, and financial benefits have contributed to their rising popularity over the past few years. Two types of energy storage devices are typically used in electric and hybrid electric designs: the “main energy system” (MES) and the “rechargeable energy storage system” (RESS). While MES increases fuel efficiency, RESS improves acceleration and regenerative braking. As the output voltage of these devices varies with load or state of charge, vehicle designers face significant challenges when integrating energy storage/supply devices with a traction drive [5]. The demand for power electronics converters has grown significantly along with the use of EVs. Power electronics converters are essential for EVs because they convert energy from a power source into a form suitable for their electric drive system. It is absurd to think EVs could run without power electronics converters. This significance has sparked an explosion in research into power electronics converters for EVs across the globe. EVs and the infrastructure that supports their charging use converters of all kinds, such as DC-DC, DC-AC, and AC-DC. In this case, DC-DC converters are more critical for integrating EV driving systems with energy sources and storage systems [6]. The required power electronics converter for an electric car and its charging infrastructure is illustrated in Figure 3.

The various EV power supply designs highlight the need for a DC/DC converter to connect the FC or battery module to the DC link. One type of power converter in electrical engineering is the DC-to-DC converter. This electric circuit momentarily stores the energy input and then releases it to the output at a different voltage. Some devices that store energy use electric fields, such as capacitors, while others use magnetic fields, such as inductors and transformers. To manage the DC-link voltage and increase the Fuel Cell voltage, a DC/DC converter is employed during fuel cell interfacing [7,8]. Several different topologies for DC-DC converters have been developed, some of which have direct energy conversion mechanisms while others do not. But, there are a few things that must be considered in the design for use in automobiles:

⮚ Slim and portable.
⮚ Very efficient.
⮚ Compact size.
⮚ EMF interference is minimal.
⮚ The current ripple is kept low by drawing from the battery or the fuel cell.
⮚ The converter exhibits a high step-up function.
⮚ Adjusting converter input voltage significantly affects DC/DC converter power flow management.

Modern electronic applications use a broad array of DC-DC converters to modify input voltages to satisfy operating needs dynamically. These converters are integral to electronic systems, which may be categorized into isolated and non-isolated varieties. To ensure that changes on the input side do not impact the output, isolated DC-DC converters utilize transformers to achieve galvanic isolation between the input and output. Because it has a ground, the converter’s input is entirely separate from the output. Depending on the setup, the output polarity can provide either positive or negative numbers [9]. Despite their superior electrical safety, isolated converters come with a price tag, a weight penalty, and many other issues, including thermal effects, core saturation, leakage inductance, dimensional constraints, and high voltage spikes in switches. On the other hand, since non-isolated DC-DC converters lack galvanic isolation, changes in the input and output are directly correlated. Despite having fewer components than their isolated counterparts, they still require careful correction to perform at their best. High-duty cycle ratios, insufficient voltage gain, and the need for additional circuitry are problems. However, non-isolated DC-DC converters perform better than their isolated counterparts in applications related to EVs. Scientists are striving to enhance the efficiency and functionality of non-isolated DC-DC converters to progress their technological capabilities. In non-isolated converters, every converter topology has its pros and cons. To illustrate, the DC/DC boost converter needs to improve regarding electrical isolation. Furthermore, the switch is subjected to intense strain due to the significant magnitude difference between the input and output. The topology is bulky and heavy and has problems with high currents and voltage ripples. A straightforward integrated multilevel DC/DC converter topology can reduce inductors’ volume and weight and increase their efficiency by lowering input and output current and voltage ripples. However, when a large voltage step-up ratio is needed, these structures fail to operate effectively [10,11].

Scientists have devoted a great deal of effort to studying DC-DC boost converters that have been customized and improved; now, they are shifting their focus to QBCs, which are converters that have a substantial gain. This tactical adjustment is the consequence of a detailed examination of the shortcomings of standard DC-DC boost converters. Although their conventional counterparts have demonstrated efficacy in some applications, they require assistance achieving high voltage gain. Designed specifically for high gain, QBC offers a novel way around these limitations [12]. The exceptional capability of QBCs to circumvent the limitations of conventional designs is a significant factor in their immense popularity. Because of their unique design, these converters minimize duty cycles while achieving significantly higher voltage improvements. QBCs are ideal for EVs because they increase range while reducing power consumption. Their high efficiency in amplifying low voltage makes them a perfect match for renewable energy sources such as solar panels and fuel cells, as well as the fluctuating power needs of EVs. The electric car industry has transitioned from traditional QBC to high-gain quadratic boost converters (HG-QBC) due to optimization, flexibility, and performance demands. Even though it can efficiently increase voltage, the quadrature boost converter might need some updates to meet the requirements of current systems [13]. To overcome these restrictions, high-gain quadrature boost converters offer several significant advantages. One important reason is the need to increase the efficiency of power conversion. High-gain quadrature boost converters are designed with this principle in mind to improve system performance and reduce energy loss during voltage boosting. The most obvious applications for this efficiency boost rely heavily on power savings, such as battery-operated devices or renewable energy systems [14].

**Figure 4:**
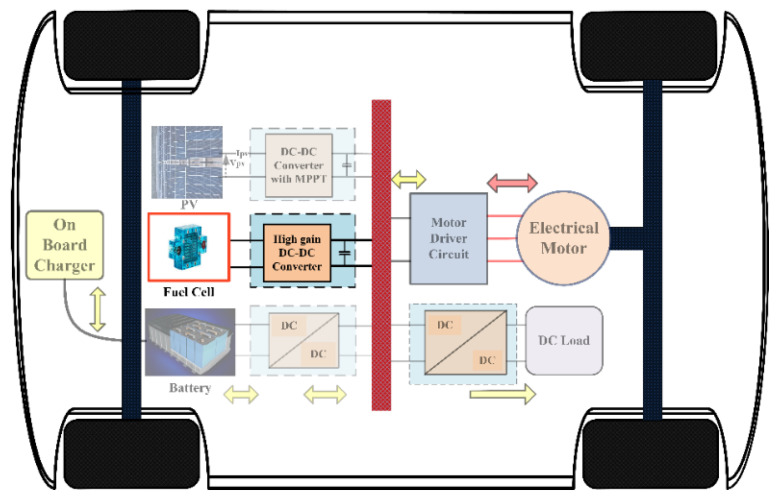
Application of high-gain converter in EV system.

The low voltage generated by the hydrogen fuel cell is converted into the high voltage needed by the high-gain DC-DC converter, as seen in Figure 4. Different configurations for non-isolated HG-QBC have been extensively discussed in the literature. There are various methods and components used in this field, such as voltage multipliers, cascade connection approaches, switching inductors, and conventional and customized QBCs. Additional techniques have been documented to enhance voltage amplification and develop novel boost converters with high gain, building upon the principles of QBC. A notable limitation of traditional QBCs and conventional boost converters is that the output voltage and the voltage stress across the switch are equal. This calls for stronger device requirements [15]. There have been various proposed topologies to tackle these issues, and among them, boost topologies based on voltage multiplier cells and switch capacitors have gained popularity for their proven practicality and effectiveness [16,17]. For high-voltage applications, the authors of [18] propose a QBC configuration that includes an inductor and a switched capacitor to achieve a higher gain. Numerous reports have been made on DC-DC converters utilizing switching capacitors [19,20]. A significant concern regarding this boost converter based on a switched capacitor voltage multiplier cell (VMC) is its high inrush current tendency. Despite its straightforward design and impressive ability to provide a substantial voltage gain while occupying minimal space, this issue must be addressed. The authors propose a modified boost converter design in [21], incorporating switching inductors and a VMC. This particular design aims to enhance efficiency and reduce stress on the capacitors. The converter can be used in applications that involve solar photovoltaics. There are various topologies proposed in [22] that utilize switched capacitors and switched inductors. An ideal high-gain DC-DC converter should possess certain characteristics for optimal performance. These include a shared ground, a consistent input current, a minimal number of components, and minimal strain on the passive components and switching devices in terms of voltage and current. The method is described in [23] with an arrangement that combines series and parallel elements in an interleaved manner. The interleaved configuration is a crucial factor in achieving high gain. However, as the number of switches increases, so does the gain. A highly efficient converter that can operate in triple-duty mode is recommended in reference [24]. However, achieving a significant gain necessitates the use of multiple switches. The voltage gain of the converter is enhanced by combining regular boost converters with Luo converters, as shown in [25]. Utilizing a significant quantity of switches enables the mitigation of voltage stress on semiconductor components. This cascaded boost converter has the potential to improve its efficiency by addressing the significant conduction losses associated with the input inductors. To minimize conduction losses, the authors of [26] suggest an enhanced cascaded boost converter. A cutting-edge design greatly minimizes the current ripple value of the input inductor. This leads to a decrease in current ripple, which in turn reduces the losses in the input inductor due to conduction. An interconnected inductor and a VMC [27]—comprised of three capacitors and two diodes—are utilized together with a standard QBC to attain a substantial voltage increase. The architecture demonstrates five different operational situations within a single switching period. Due to the intricate complexity of the system, developing the controller for a switching regulator using the topology above will be quite challenging. To meet the requirements for fuel cell and electric car applications, DC-DC converters need to possess a high gain, minimal stress across capacitors, and a continuous input current [28,29]. In [30], a method called the voltage-lifting (VL) approach is employed to enhance the voltage and gain of the QBC. A highly effective approach to improve the voltage gain of the converter is by utilizing the voltage-lift cells. Given its superior quality, a converter can achieve a significant increase in voltage while keeping costs low and power density minimal. One additional benefit of the VL techniques is the reduction in voltage and current ripple.

Additionally, there is a wide variety of high-gain DC-DC converters available, each with its own unique design, set of features, and applications. Numerous scholars and companies are currently dedicated to addressing these limitations. Given the current state of the market for EVs, this field of study is receiving significant attention. Prior to its practical application in e-mobility, the high-gain DC-DC converter needs to undergo enhancements and careful considerations from various perspectives. When developing a high-gain DC-DC converter for real-world EV systems, it is crucial to carefully evaluate various parameters. These include input current, voltage and current stresses on devices and components, waveforms of these parameters, and the overall number of components.

## 2. Quadratic Boost Converter

**Figure 5:**
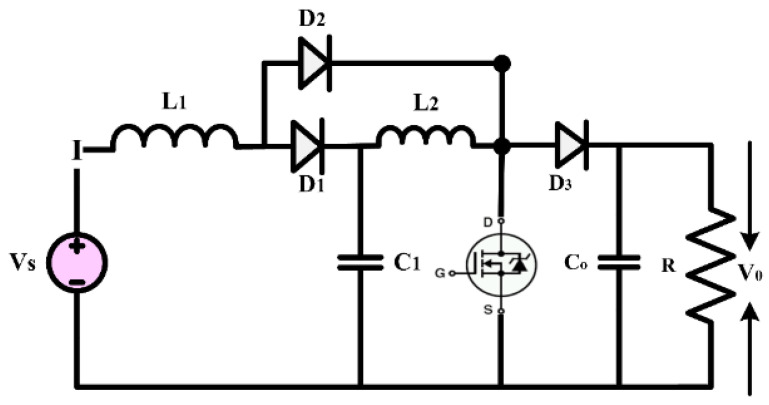
Circuit diagram of quadratic boost converter.

The conventional QBC is utilized for in-depth analysis, and the structure of the converter is depicted in Figure 5. Compared to a traditional boost converter, a QBC has better gain and can draw a current without ripples from sources that do not produce a pulsing input current. As a result, the sources will be more reliant on each other [31,32,33]. The increased interdependence can enhance the overall performance and efficiency of the system. In addition, the QBC’s capability to maintain a smooth current flow makes it ideal for use when a reliable and steady power source is necessary. This is particularly important for delicate electronic devices or precise instruments.

A QBC circuit topology included a single switch, two inductors, a capacitor, and three diodes. By activating the switch (S), the input source charges the inductor $L_{1}$, and by turning on Capacitor $C_{1}$, the inductor $L_{2}$ is charged. The load is responsible for discharging the stored energy in the inductor. In the absence of the switch (S), the input source and capacitor $L_{1}$ are simultaneously used to charge capacitor $C_{1}$. Continuous conduction mode (CCM) displays two modes based on the converters’ modes of operation, while discontinuous conduction mode (DCM) displays three modes.

**Figure 6:**
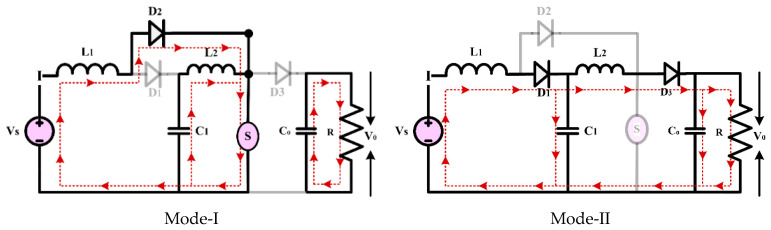
Mode of operation of the conventional quadratic boost converter.

CCM Mode of Operation:

Mode 1: the equivalent circuit of the converter in CCM is shown in Figure 6. This mode of operation is present when switch S is on from 0 < t < $dT_{S}$. In this mode, the Diode $D_{2}$ is the forward bias condition, and the diodes $D_{1}$ and $D_{3}$ are the reverse bias condition. The inductor $L_{1}$ is charged from the input source and $L_{2}$ is charged from the capacitor $C_{1}$. The capacitor $C_{0}$ is deliver the power to load. In this mode, the voltage across the inductor $L_{1}$ is equal to input voltage $V_{S}$ and voltage across the inductor $L_{2}$ is equal to voltage across the capacitor $C_{1}$.

(1) $$V_{0}=V_{Co}$$

Mode 2: the equivalent circuit of the converter in mode 2 is presented in Figure 6. in this mode of operation, the switch S is off in the period of $dT_{S}$ < t < (1 − d)$T_{S}$. The diodes $D_{1}$ and $D_{3}$ behave as a forward biased and the diode $D_{2}$ behaves as a reverse biased. The inductors $L_{1}$ and $L_{2}$ are discharged using the load and $C_{0}$. The capacitor $C_{2}$ is charged using the inductor $L_{1}$ and the input source.

The voltage gain of the converter in CCM is calculated using the volt- second balance of the inductors $L_{1}$ and $L_{2}$. The average voltage of the inductor is zero.

When switch *S* is ON,

(2) $$V_{L_{1}}=V_{S}$$

(3) $$V_{L_{2}}=V_{C_{1}}$$

When Switch *S* is OFF,

(4) $$V_{L_{1}}=V_{S}-V_{C_{1}}$$

(5) $$V_{L_{2}}=V_{C_{1}}-V_{C_{0}}$$

Volt-second balance across the inductor $L_{1}$,

$$dV_{s}+\left(1-d\right)\left(V_{S}-V_{C_{1}}\right)=0$$

(6) $$V_{C_{1}}=\frac{V_{s}}{\left(1-d\right)}$$

Volt-second balance across the inductor $L_{2}$,

$$d(V_{C_{1}})+\left(1-d\right)\left(V_{C_{1}}-V_{C_{0}}\right)=0$$

(7) $$V_{C_{0}}=\frac{V_{C_{1}}}{\left(1-d\right)}=\frac{V_{s}}{{\left(1-d\right)}^{2}}$$

Putting the value of Equation (7) in Equation (1),

(8) $$V_{O}=\frac{V_{s}}{{\left(1-d\right)}^{2}}$$

The gain of the purposed converter in CCM is,

(9) $$M=\frac{V_{0}}{V_{S}}=\frac{1}{{\left(1-d\right)}^{2}}$$

**Figure 7:**
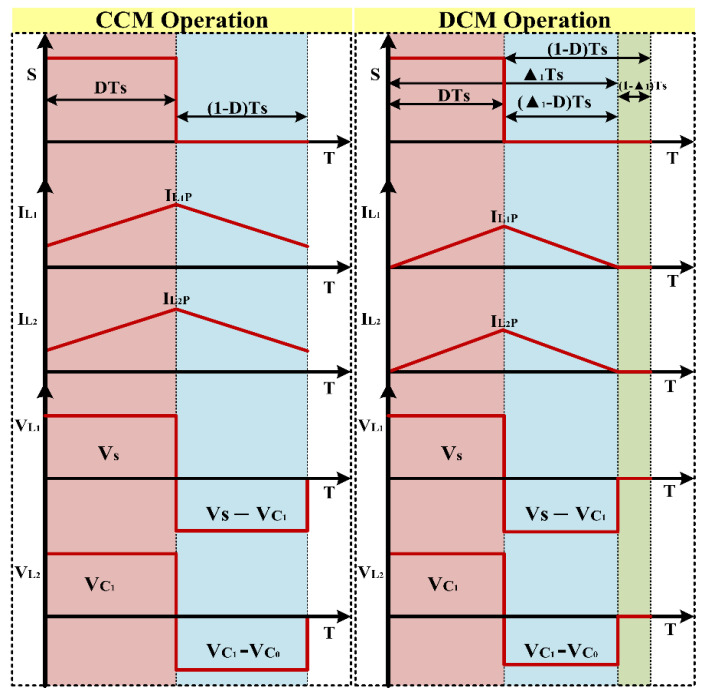
Theoretical waveform of QBC in CCM and DCM mode.

DCM Mode of Operation:

The QBC’s analytical waveform during a DCM operation is illustrated in Figure 7. In a DCM state, there are three possible modes of operation.

Mode 1: when switch S is on from 0 < t < $dT_{S}$. Where the inductor $L_{1}$ current started from zero and reached up to $I_{L_{1}}$ at $t=dT_{S}$ and inductor $L_{2}$ also start from zero and reaches up to $I_{L_{2}}$ at $t=dT_{S}$. There, for the mode of operation one in DCM, it is like mode one in CCM.

(10) $$V_{L_{1}}=V_{S}$$

(11) $$V_{L_{2}}=V_{C_{1}}$$

Mode 2: in this mode switch s is turned off from $dT_{S}$ < t < ${∆}_{1}-D$ and the inductor current starts decreasing and reaches zero.

(12) $$V_{L_{1}}=V_{S}-V_{C_{1}}$$

(13) $$V_{L_{2}}=V_{C_{1}}-V_{C_{0}}$$

Mode 3: mode 3 starts when the inductor current reaches zero, from ${∆}_{1}$ to maintain zero until the next cycle starts. In this mode, the power delivered from the input supply is zero and the output power is delivered using capacitor $C_{0}$.

(14) $$V_{C_{0}}=V_{0}$$

The gain of the purposed converter in DCM is,

(15) $$M=\frac{V_{0}}{V_{S}}=\frac{{{∆}_{1}}^{2}}{{\left({∆}_{1}-d\right)}^{2}}$$

Based on the converter’s switching behaviors, two modes are provided: CCM and DCM. The analytical waveform of the suggested converter is shown in Figure 7.

### Experimental Analysis of Quadratic Boost Converters

**Figure 8:**
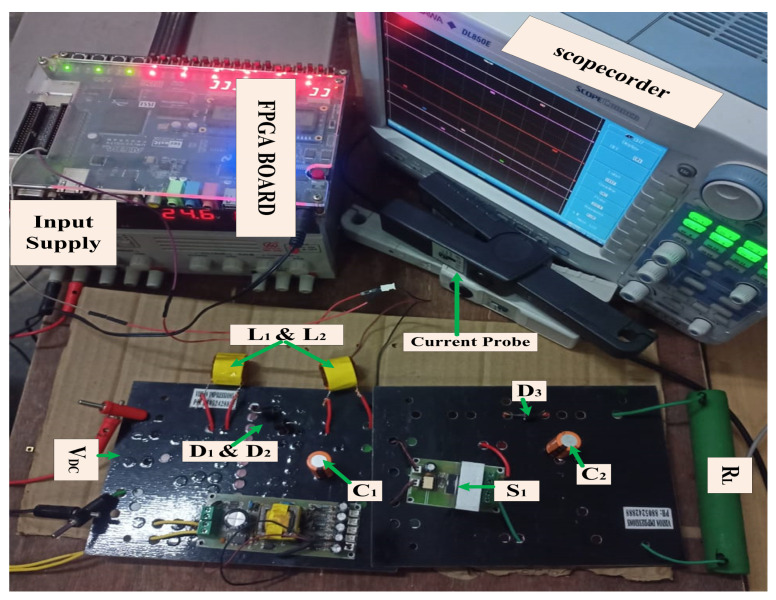
Hardware setups of conventional quadratic boost converter.

**Figure 9:**
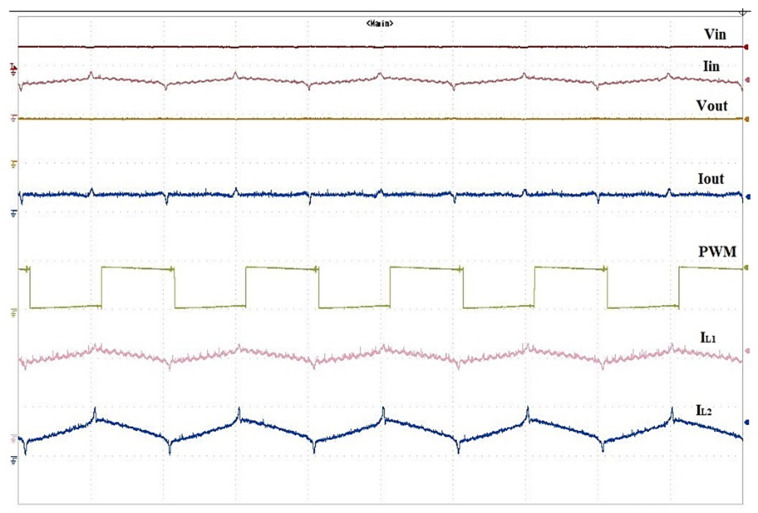
Output waveform of the conventional quadratic boost converter.

Figure 8 illustrates the hardware test bench for conventional QBC. The construction of a 150 W quadratic boost converter circuit involves the use of two inductors, two capacitors, three diodes, and one switch. The values of the inductors L_1_ and L_2_ are 0.3 μh and 0.47 μh, respectively. Capacitors C_1_ and C_2_ have respective values of 22 μf and 10 μf. The results of the conventional QBC through the experimental setup are highlighted in Figure 9. The specifications detail an input voltage of 25 V, an output voltage of 100 V, a switching frequency of 50 KHz, a load resistance of 230 Ω, and a duty cycle (D) set to 0.5. After analyzing the test results, it was noted that the QBC showed a voltage gain (M) of four times when running at a 50% duty cycle. Based on the results, it is evident that maintaining the traditional QBC duty cycle is essential for achieving the maximum voltage gain.

QBC is an essential component in various systems, such as EVs, battery charging, renewable energy generation, internet connectivity, patient treatment, communication, satellite and aircraft power, and LED systems [34,35]. Through improvements in electricity transmission, the driving range and efficiency of EVs are expanded. Ensuring the seamless integration and storage of renewable energy sources into the grid is achieved through the optimization of solar panel energy harvesting. They offer voltage levels that enable charging systems to charge batteries efficiently and rapidly. By carefully managing voltage levels in different subsystems, they guarantee reliable and efficient power distribution in aerospace and satellite systems. Devices on the Internet of Things, like sensors, microcontrollers, and communication modules, maintain stable voltage levels, resulting in extended battery life for battery-operated products. They ensure the reliability and accuracy of medical equipment by managing and improving voltage control. They are responsible for managing and regulating power in telecommunications infrastructure by adjusting voltages to meet the needs of communication systems. They offer dependable and eco-friendly lighting solutions by managing and improving voltages in LED drivers utilized in LED lighting systems. QBCs play a crucial role in numerous systems because they can effectively regulate power and convert voltage. They play an essential role in the ever-changing realm of electric and electronic systems, thanks to their ability to efficiently boost voltage levels.

By comparing the QBC to interleaved, cascade, and conventional boost converters, one can gain a comprehensive understanding of its various features, capabilities, and limitations. The chart provides a comprehensive display of critical aspects such as control strategies, efficiency metrics, concerns about output ripple and noise, and voltage gain characteristics. Moreover, it clarifies if the parameters are suitable for various applications. The QBC is a recent entrant in the high-gain DC-DC converter market due to its unique voltage gain characteristics. Due to its unique ability to alter the relationship between input and output voltage, the QBC shows potential in applications that demand specific voltage profiles. In contrast to the linear voltage gain commonly employed in boost converters, this operates differently. An evaluation is conducted on the effectiveness and ability to handle the power of interleaved boost converters. These converters have become widely recognized for their improved reliability and reduced output ripple. Cascade boost converters offer a fascinating option for high-voltage situations, thanks to their ability to stack voltages multiplicatively. To assess the novel QBC, it is worth considering its performance compared to the well-established traditional boost converters [36,37]. The chart provides a clear visual representation of the advantages of various converters. This can assist power electronics researchers, engineers, and practitioners in making well-informed decisions tailored to their specific application requirements. This comprehensive comparison adds to the ongoing discussion of advanced DC-DC converters by shedding light on their efficiency and usefulness in various situations. The Comparative analysis of different types of boost converter topologies is presented in Table 1.

The analysis and comparison of the QBC with cascade, interleaved, and traditional boost converters in the above chart has brought attention to the urgent need to develop high-gain DC-DC converters. The losses associated with the inductor, filter capacitor, and main switch in a conventional DC-DC boost converter limit the achievable voltage gain. Given the significant voltage stress across the switching device, it is crucial to select a switching device with a high voltage rating. Despite their advantages in reducing input current ripple, interleaved converters face challenges when it comes to handling low voltage gains and require a multitude of components, resulting in larger sizes and decreased efficiency. One popular technique for obtaining a modest voltage out of typical DC-DC converters is cascading. By increasing the number of switches in a cascaded power converter, it becomes possible to achieve an average ratio of voltage conversion. To optimize the margin, the input supply is directly transmitted to the first stage of the cascaded converter, where the duty cycle is increased to raise the voltage. As the duty cycle decreases in succeeding stages, the impact of switching losses becomes less significant. There is a high number of switches, complex circuitry, and control switches present at every level. The extensive collection of inductors, diodes, capacitors, and active switches lead to a decrease in durability, while still maintaining a high voltage conversion ratio. The QBC partially mitigates the limitations of these converters. The QBC method is implemented in a step-by-step manner using a single switch and a set of uncontrolled switches (diodes). There is still a notable drawback that remains, as the overall gain of the QBC is determined by multiplying the voltage gains of each stage. The QBC’s fourth-order mechanism introduces additional complexity and reduces efficiency, while the regulated switch experiences significant voltage stress, which is its main drawback. The overall output voltage is equivalent to the voltage strain across the controlled switch. As a result, a switch with a higher rating is required, leading to an increase in the price of the converter. Various techniques are employed in converters to produce a high voltage, minimize voltage strain, and enhance efficiency. These strategies involve utilizing switched capacitors (SC), switched inductors (SI), or a combination of the two. There is a growing demand for power electronics technologies that are effective and adaptable, which has led to the need for innovative architectures that can greatly increase voltage upscaling. The demand for high-gain converters is increasing due to the rapid expansion of various technical applications, including renewable energy systems and electric automobiles.

## 3. High-Gain Quadratic Boost Converter Topology

**Figure 10:**
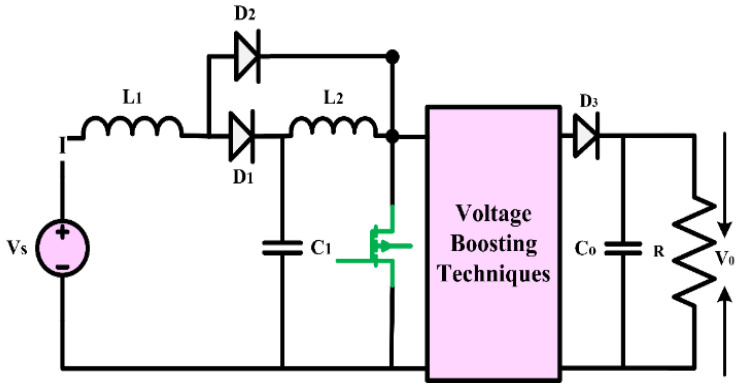
Design architecture of a high-gain converter utilizing a QBC.

The high-gain quadratic boost converter (HG-QBC) architecture shown in Figure 10 is widely recognized as a crucial component in the transformation of electric propulsion system performance and efficiency standards. The fundamental requirement for enhanced voltage conversion is addressed by HG-QBCs, making them indispensable. This, in turn, enables a more efficient and seamless energy flow within EVs. This advancement represents a deliberate move towards aligning power electronics with the ever-changing world of sustainable transportation. HG-QBCs have a unique quadratic input–output relationship that allows them to efficiently convert voltages, surpassing the capabilities of traditional boost converters. Furthermore, this enhancement plays a crucial role in improving the overall efficiency of electric propulsion systems. It also plays a significant part in supporting the electric mobility ecosystem’s sustainability objectives [38,39]. The illustration below showcases the HG-QBC topology, highlighting its intricate architecture and its potential to bring about a significant transformation in energy conversion for electric automobiles.

HG-QBCs are crucial in the quest for environmentally friendly EVs because of their exceptional quadrature boost capabilities. Scientists are exploring intermittent and unpredictable energy sources, such as solar power and fuel cells, as potential power sources for EVs, driven by the goal of sustainable development. Nevertheless, ensuring a consistent energy supply for EVs remains a formidable task, given these sources’ inherent intermittency and unpredictability. To understand HG-QBCs comprehensively, it is essential to grasp their functioning, operation, efficiency testing, and compatibility with different renewable energy sources. Academics have made significant design optimizations to ensure that the HG-QBC can be charged with various energy sources, making it suitable for electric mobility in different input scenarios. These converters need to meet specific performance criteria to guarantee reliability, lifespan, and compliance with industry requirements [40]. Thanks to advancements in this field, specific standards have been established to regulate the practical application of HG-QBCs. These standards ensure their seamless integration with other energy sources, particularly in the development of EV. Research is focused on developing standards for integrating HG-QBCs with various energy resources. However, it is still being determined if these standards will be fully implemented in real-world scenarios and if they will be reliable and durable. HG-QBCs are highly regarded for their essential role in advancing EV sustainability, owing to their exceptional quadrature boost capabilities. Researchers are investigating intermittent and unpredictable energy sources, like solar power and fuel cells, as potential power sources for EV, driven by the aim of sustainable development. However, guaranteeing a reliable energy supply for electric cars is still a significant challenge, considering these sources’ natural fluctuations and uncertainties. To fully comprehend HG-QBCs, it is crucial to thoroughly understand how they work, their operation, efficiency testing, and their compatibility with various renewable energy sources. Researchers have implemented essential design improvements to ensure that the HG-QBC can be charged using multiple energy sources, making it ideal for electric mobility in various input scenarios. These converters must satisfy specific performance criteria to ensure reliability, longevity, and adherence to industry standards [40]. Recent progress in this area has allowed for the establishment of precise guidelines to control the real-world use of HG-QBCs. These standards guarantee a smooth integration with other energy sources, especially in advancing EVs. Our aim is to establish standards for seamlessly integrating HG-QBCs with different energy resources. However, it remains to be seen whether these standards will be effectively implemented in practical situations and whether they will demonstrate reliability and durability.

In this section, a few high-gain prior-art converters based on QBC topology are examined. It also describes other boosting topologies, such as voltage lifting, voltage doubler circuits, switch capacitors, switch inductors, and switch capacitors and inductors. These topologies are typically combined with a QBC to achieve high-voltage gain. These boosting methods can be divided into three groups: switch capacitors, switch inductors, and hybrid switch capacitors and inductors.

### 3.1. Switch Capacitor-Based High-Gain Quadratic Boost Converters

In this configuration, switch capacitors are used in conjunction with either conventional QBCs or modified QBCs to create the innovative high-gain converter. The circuit diagram of a conventional QBC is presented in Figure 5. The uncontrolled or controlled switched capacitors are connected in a specific way with QBCs so that they store and release energy in each cycle, which effectively enhances the gain of the converters. This section presents two high-gain converters that utilize the switch capacitor topology. Furthermore, two distinct modes of operation, Mode-I when switch S is turned ON and Mode-II when switch S is closed, are taken into consideration.

#### 3.1.1. High-Gain Quadratic Boost Converters I (HG-QBC I)

**Figure 11:**
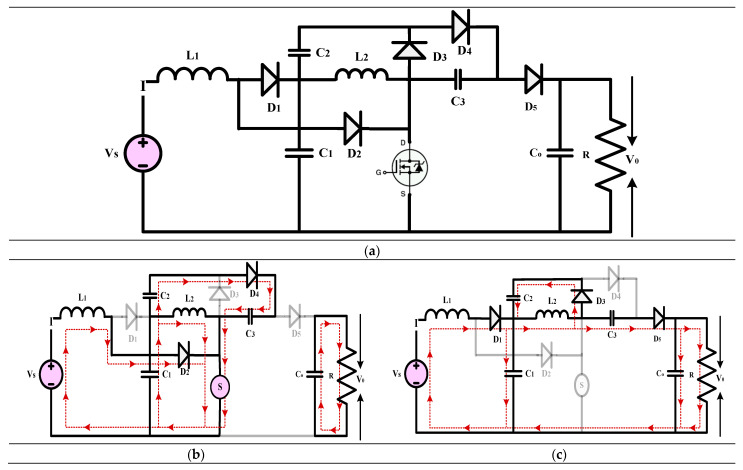
(**a**) Circuit diagram of QBC based high-gain converter (**b**) Mode-I Operation (**c**) Mode-II Operation.

The high-gain converter illustrated in Figure 11, disclosed in [41], introduces a revised voltage-lift cell for a QBC to enhance voltage gain and reduce switch voltage stress. An analysis and comparison are performed to understand the operating principle of the converter under consideration by comparing it with similar single-switch high-gain converters. A validation study is conducted to verify and validate the findings of the theoretical analysis. Compared to the standard voltage-lift cell, the modified version, with an extra capacitor and diode, effectively reduces switch voltage stress and enhances voltage gain. The study primarily analyzes the waveforms and modes of operation of the suggested QBC. This QBC utilizes a modified voltage-lift cell and operates in continuous conduction mode. Based on a comparison study with existing high-gain converters, the recommended converter achieves the lowest switch voltage stress and the most significant voltage gain for a specific duty ratio. Confirming the theoretical analysis, the experimental results demonstrate the effectiveness of the suggested converter. Based on the results, it is evident that the proposed converter with the improved voltage-lift cell outperforms conventional single-switch high-gain converters. This makes it an ideal choice for applications requiring a significant voltage increase.

(16) $$Gain=\frac{V_{0}}{V_{S}}=\frac{2}{{\left(1-D\right)}^{2}}$$

#### 3.1.2. High-Gain Quadratic Boost Converters II (HG-QBC II)

**Figure 12:**
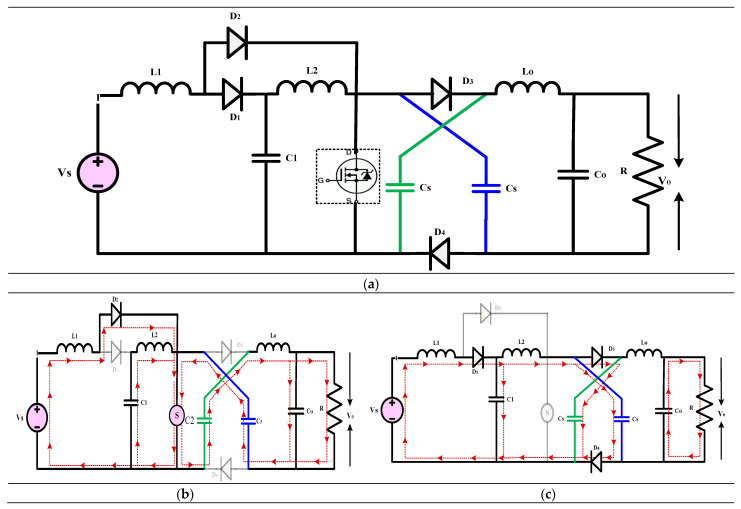
(**a**) Circuit diagram of QBC based high-gain converter (**b**) Mode-I Operation (**c**) Mode-II Operation.

The high-gain converter shown in Figure 12 is described in [42]. The authors investigate the expanding demand for high-voltage gain DC-DC switching converters across various sectors, including renewable energy, healthcare, manufacturing, and transportation. Regarding conventional QBCs, the authors offer a solution to the issue of increased voltage stress on the active and passive switches. The combination of an output filter with a voltage multiplier cell accomplishes this. To make the converter more accurate, the paper gives formulas for the inductor and capacitor voltages and currents and the corresponding ripples. A methodical strategy for creating an average current-mode controller is suggested to examine the converter’s dynamic behavior comprehensively. This method produces a linear averaged, nonlinear averaged, and bilinear switched model. According to the findings of the tests, the switching regulator is quite robust. This demonstrates a prototype that can provide 220 V and 300 W power output. Both the input and output currents of the proposed converter are stable, and it is incredibly efficient. This characteristic makes it possible to use a broad range of renewable energy sources, increasing the longevity of those sources and the equipment that uses them.

(17) $$Gain=\frac{V_{0}}{V_{S}}=\frac{\left(1-D\right)}{{\left(1-D\right)}^{2}}$$

### 3.2. Switch Inductor Based High-Gain Quadratic Boost Converters

In this configuration, switch inductors are used with either a traditional QBC or a modified QBC to create a state-of-the-art high-gain converter. To increase the converters’ gain, a certain way of connecting the controlled or uncontrolled switching inductors to the QBC allows them to store and release energy throughout each cycle. In this section, two high-gain converters that utilize the switch inductors’ topology are described. In addition, two different modes of operation, Mode-I when switch S is ON and Mode-II when S is closed, are discussed.

#### 3.2.1. High-Gain Quadratic Boost Converters III (HG-QBC III)

**Figure 13:**
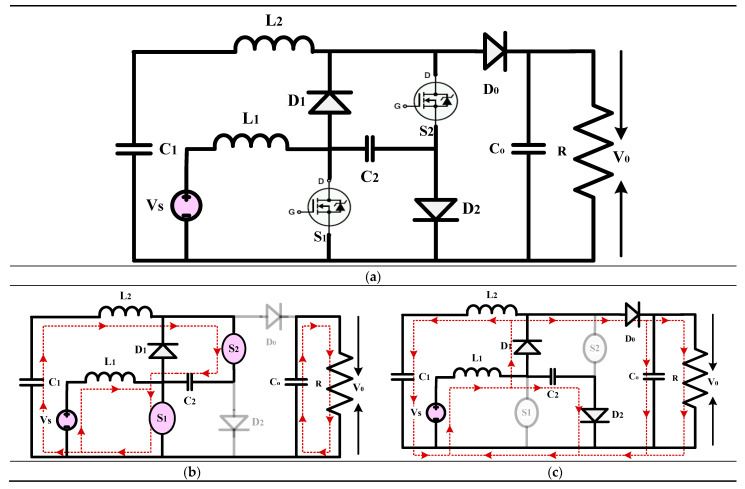
(**a**) Circuit diagram of QBC based high-gain converter (**b**) Mode-I Operation (**c**) Mode-II Operation.

The high-gain converter represented in Figure 13 is explained in [43]. The article introduces a novel high-voltage gain converter that takes advantage of the asymmetric input voltage of inductors. The converter showcases impressive power density, a favourable output, and a consistent input, making it an ideal choice for renewable energy applications. The efficiency, small-signal analysis, practical voltage gain, steady-state performance, and operating theory of the converter have been analyzed. A comprehensive analysis is conducted on various components to assess and compare the current converters. This includes evaluating the voltage gain, effectiveness index, stress on power devices (both voltage and current), switching device ratings per unit, and other relevant factors such as output polarity and the availability of common ground. The suggested converter is compact and highly efficient, with a lower power rating for the switching device and an enhanced effectiveness index. A laboratory prototype with a power output of 150 W is being used for experiments to confirm its functionality. The converter comprises three diodes, two inductors, three capacitors, a load resistance, and two active power semiconductor switching switches that operate in synchronization. The experiment’s findings align with the theoretical predictions for the converter, and the achieved voltage gain closely resembles the ideal but imperfectly implemented voltage gain.

(18) $$Gain=\frac{V_{0}}{V_{S}}=\frac{\left(1-D\right)}{{\left(1-D\right)}^{2}}$$

#### 3.2.2. High-Gain Quadratic Boost Converters IV (HG-QBC IV)

**Figure 14:**
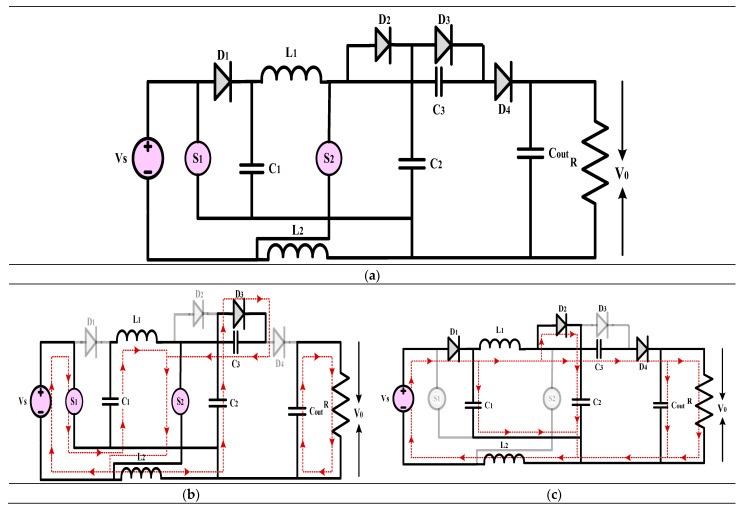
(**a**) Circuit diagram of QBC based high-gain converter (**b**) Mode-I Operation (**c**) Mode-II Operation.

The high-gain converter shown in Figure 14 is explained in [44]. This article introduces a DC-DC converter that achieves a substantial voltage gain while operating without a transformer and is suitable for low to medium-power applications. The miniaturization of the converter is achieved using only two inductors, which nonetheless permit a broad range of duty ratio modifications to attain the target output voltage. Using low voltage-rated components helps to lessen voltage stress across switches, which is what makes this converter unique. It also boasts a robust quadratic gain. It is easy to control the input current because it is continuous. It is possible to use the converter in either of two modes: Mode I or Mode II. The typical voltage stress of the proposed converter switches is lower than that of alternative topologies. The converter’s functional model was created in a controlled environment utilizing the power circuit board approach. This converter can handle input voltages between 12 and 20 volts and provides up to 200 watts of electricity, according to its specifications. Its switching frequency is 50 kHz. The converter cannot function without the constant current mode (CCM) features, which include continuous inductor currents and low voltage stress across the switches. The thermal model shows an outstanding efficiency of 94.5% when run on 24 volts. In addition, it uses less than 91% energy for the whole 200-watt input power cycle, which is rather impressive.

(19) $$Gain=\frac{V_{0}}{V_{S}}=\frac{3-D^{2}}{{\left(1-D\right)}^{2}}$$

### 3.3. Hybrid Switch Capacitor and Inductor-Based High-Gain Quadratic Boost Converter

In this configuration, a combination of switch capacitor and inductor is utilized alongside either a conventional QBC or modified QBC to develop a cutting-edge high-gain converter. The uncontrolled or controlled hybrid switch capacitor and inductor are interconnected in a specific manner using a QBC to efficiently store and release energy in each cycle, thereby significantly boosting the converters’ gain. Here, six examples of high-gain converters that make use of the hybrid switch capacitor and inductor topology are analyzed. Additionally, two different operating modes, Mode-I for when switch S is in the on position and Mode-II for when switch S is closed, are taken into consideration. These two modes of operation offer flexibility in managing the energy flow and maximizing the high-gain converter’s performance. In many applications where large gains are essential, the converters that are being introduced provide dependable and effective solutions.

#### 3.3.1. High-Gain Quadratic Boost Converters V (HG-QBC V)

**Figure 15:**
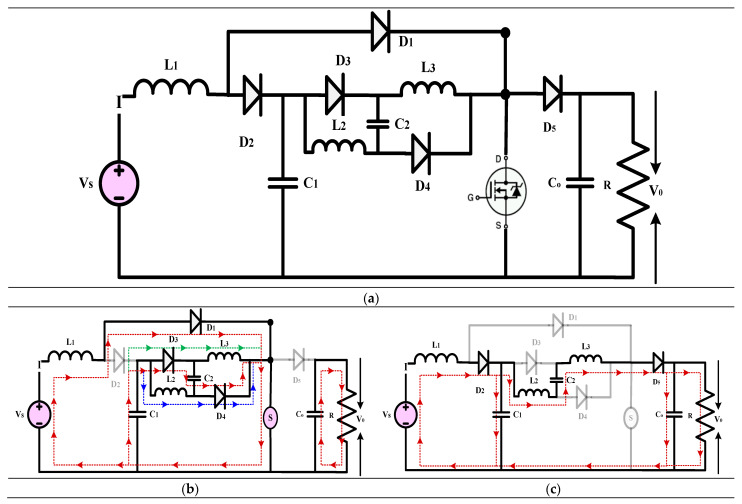
(**a**) Circuit diagram of QBC based high-gain converter (**b**) Mode-I Operation (**c**) Mode-II Operation.

The high-gain converter shown in Figure 15 is derived from [45], which explores a DC-DC boost converter with a quadratic voltage gain specially tailored for medium- and low-power applications. The design of the converter incorporates a single-stage, non-isolated configuration. The recommended converter provides a much higher level of efficiency when compared to a typical QBC. It accomplishes this through a streamlined design incorporating just one switch, making implementation easier. This product is highly compatible with renewable energy sources because of its continuous current mode operation and lack of a linked inductor. Compared to other currently available topologies, the suggested converter showcases exceptional performance in terms of efficiency and voltage gain, especially in non-isolated scenarios. This study analyzes the findings from a simulation, experimental data, and comparisons with other converters. It also covers expressions for steady-state operating, efficiency, and voltage gain, considering nonidealities. The efficiency of the suggested converter has been verified to be over 88% through extensive simulations and experiments.

(20) $$Gain=\frac{V_{0}}{V_{S}}=\frac{2}{{\left(1-D\right)}^{2}}$$

#### 3.3.2. High-Gain Quadratic Boost Converters VI (HG-QBC VI)

**Figure 16:**
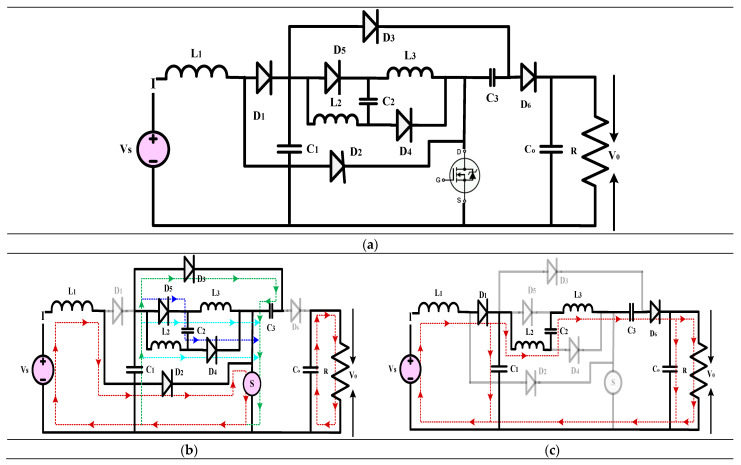
(**a**) Circuit diagram of QBC based high-gain converter (**b**) Mode-I Operation (**c**) Mode-II Operation.

The high-gain converter depicted in Figure 16 [46] is a novel non-isolated DC-DC converter with superior voltage gain and reduced component stress. Its steady current output and simplified switches demonstrate outstanding performance in microgrids. The authors investigate the converter’s operation under steady-state settings and compare its efficiency to existing high-gain topologies. The PLECS program rates the converter’s power loss and efficiency by incorporating the switching characteristics in the datasheet. The procedure starts with developing a lab hardware model and testing the outcomes through simulation. The converter’s gain, around 2.5 times more than a typical QBC, is based on the Volt-Sec balancing theory. The proposed topology offers a notable benefit for duty ratios between 0.2 and 0.8 among non-discrete topologies. Considering the operation of the diodes and switching losses determines the converter’s efficiency. To cut overall switch losses by around 18%, it is possible to use diodes or Schottky diodes with lower cut-in voltages. This converter displays a peak efficiency of over 90% at 16 V and 80 W, which is rather excellent. Therefore, it is a perfect choice for high-efficiency and power-density applications. Its compact size and advanced control features make it suitable for various applications.

(21) $$Gain=\frac{V_{0}}{V_{S}}=\frac{\left(3-D\right)}{{\left(1-D\right)}^{2}}$$

#### 3.3.3. High-Gain Quadratic Boost Converters VII, VIII, and IX (HG-QBC VII, VIII, and IX)

**Figure 17:**
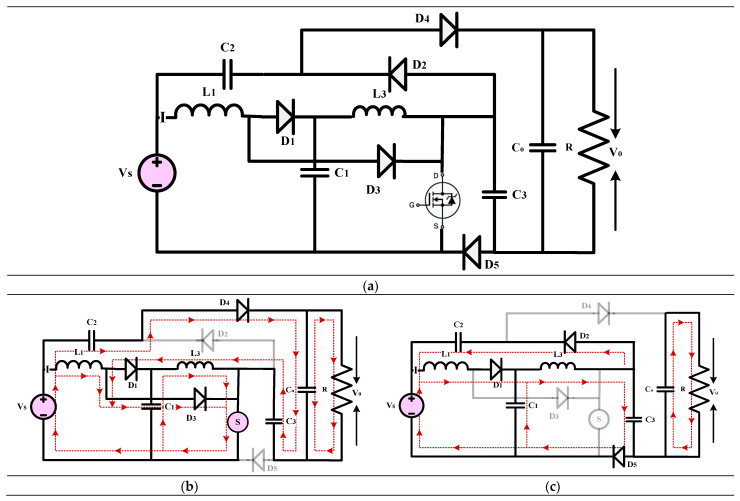
(**a**) Circuit diagram of QBC based high-gain converter (**b**) Mode-I Operation (**c**) Mode-II Operation.

**Figure 18:**
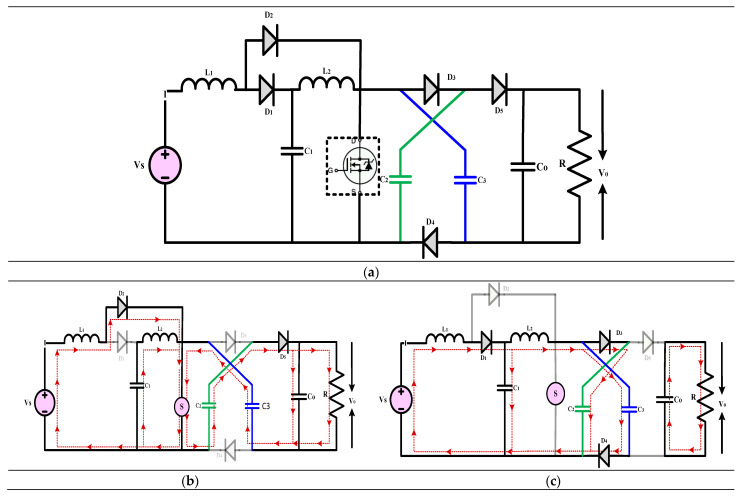
(**a**) Circuit diagram of QBC based high-gain converter (**b**) Mode-I Operation (**c**) Mode-II Operation.

**Figure 19:**
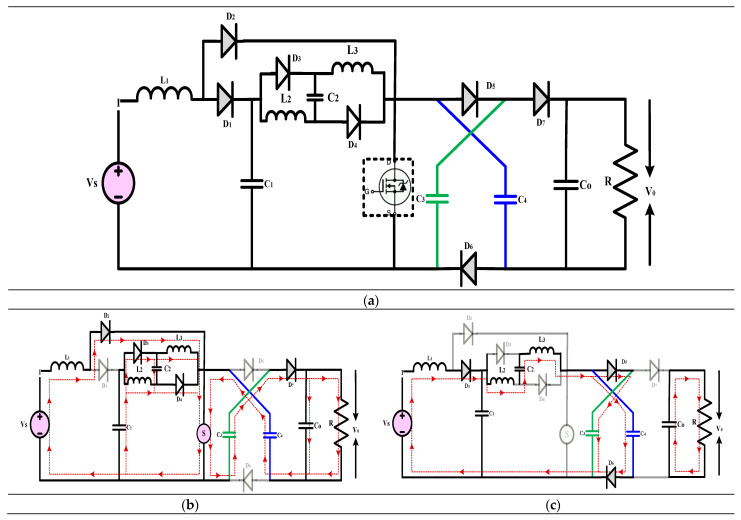
(**a**) Circuit diagram of QBC based high-gain converter (**b**) Mode-I Operation (**c**) Mode-II Operation.

Figure 17, Figure 18 and Figure 19 illustrate the high-gain converter disclosed in [47]. This work introduces three different non-isolated QBC topologies, all of which use a single switch to provide a high voltage gain with little voltage stress on the switches. These topologies use VMC, which consists of capacitors and switching inductors, to boost the converter’s voltage and gain. To calculate the converter’s non-ideal voltage gain, elements such as the parasitic capacitance, ON-state resistances of the switches and diodes, and continuous conduction mode are considered. While doing the efficiency study, the PLECS program considers the conduction and switching losses of the passive and switching parts. To ensure the functionality of the converters that have been described, an experimental prototype is developed and extensively tested. To keep the DC-link voltage stable, these converters, when positioned at the inverter’s front end, work great for microgrids’ medium power applications. The article showcases a variety of QBCs that enhance the voltage gain using switching capacitors. Comparing the recommended converter-I to the regular QBC, it is evident that the latter has a higher gain and less voltage stress across the output voltage. This method, which requires only one switch and provides a constant input current, is ideal for microgrids and solar photovoltaics use. A single switch connects the two separate DC-DC converters that make up Converters-II and III. In addition to an output capacitor, each converter has four capacitors and five diodes. The suggested topology relies on an input-side inductor to maintain a constant current state with little variation. In comparison to standard QBCs, the output voltage of Converter-III is four times higher thanks to the use of two voltage multiplier cells and a switching inductor boost cell, which are upgrades over Converter II. The proposed DC-DC converters were evaluated regarding current topologies using criteria such as component count, voltage gain, current stress, and switch voltage stress. Converters I and II use a smaller number of components in comparison to the other topologies in their reference. While all three converters achieved voltage gains, Converter III was the least efficient, while Converters I and II were the most efficient. Converter II is the most effective choice among the three recommended converters, as it has high efficiency and less stress from the voltage and current. Presenting these three novel high-gain DC-DC converter topologies, this work concludes that they are all superior for duty-gain operation in renewable power applications. These converters’ low voltage stress, high efficiency, and significant voltage gains have earned them widespread acclaim. The experimental results validate the usefulness of the proposed converters.

(22) $$Gain=\frac{V_{0}}{V_{S}}=\frac{2}{{\left(1-D\right)}^{2}}$$

(23) $$Gain=\frac{V_{0}}{V_{S}}=\frac{2}{{\left(1-D\right)}^{2}}$$

(24) $$Gain=\frac{V_{0}}{V_{S}}=\frac{4}{{\left(1-D\right)}^{2}}$$

#### 3.3.4. High-Gain Quadratic Boost Converters X (HG-QBC X)

**Figure 20:**
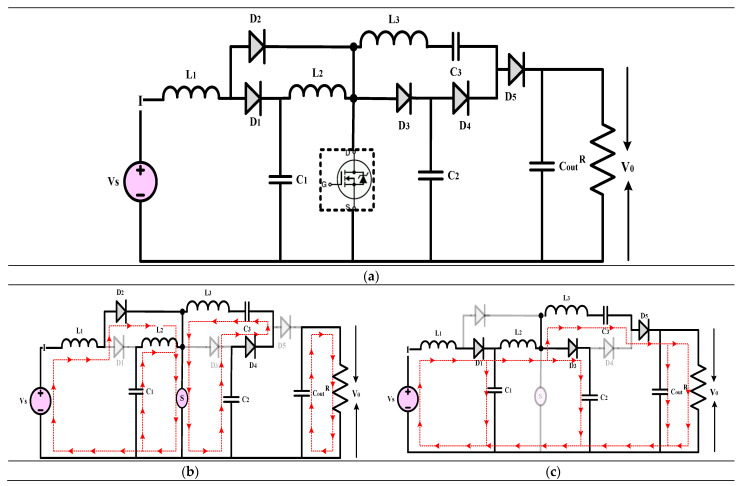
(**a**) Circuit diagram of QBC based high-gain converter (**b**) Mode-I Operation (**c**) Mode-II Operation.

The high-gain converter illustrated in Figure 20 [48], derived by integrating a VMC and a QBC, is a revolutionary design for a DC-DC converter. Although the converter’s switch use factor is significant, the voltage stress on the semiconductor devices is modest. The voltage stress of the VM cell is what defines its excellence. This converter has the same parts as any other voltage-lift converter. An experimental 40 W model is built to confirm the practicality and accuracy of the theoretical computations. The prototype receives 12 V as input and produces 96 V as output. Renewable energy systems, whether connected to the grid or not, rely on high-voltage-gain DC-DC conversion, and this research intends to develop new topologies for this process. The operating frequency, duty cycle, number of multiple cells, and output current are essential factors that significantly impact the design of a converter’s capacitor. Examining four different converters utilizing voltage gain and voltage stress as metrics, compared to the proposed 89-component, non-isolated high-gain DC-DC converter. The voltage stress on the switch and output diode is coupled with a duty cycle of 0.3, 0.5, or 0.7 in this converter. Consequently, it obtains a 2.04 W voltage gain. The voltage conversion ratio dwarfs the line voltage, which boasts a robust static gain. Adding more multiplier cells reduces the voltage applied to semiconductor devices, which protects switches and output diodes from damage. The number of cells directly affects this. A voltage multiplier cell and a QBC are included in the converter’s architecture to produce an ideal input current for fuel cell applications. A current free of ripples is made because of this. A simple control circuit and one active switch are all needed to get things going. Operating at half-load, the converter obtains an efficiency rating of 88% and boasts a user-friendly design.

(25) $$Gain=\frac{V_{0}}{V_{S}}=\frac{2}{{\left(1-D\right)}^{2}}$$

#### 3.3.5. High-Gain Quadratic Boost Converters XI (HG-QBC XI)

**Figure 21:**
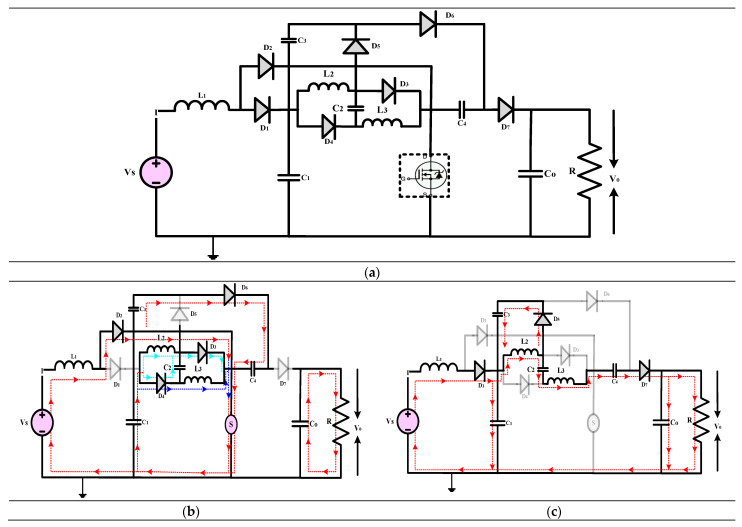
(**a**) Circuit diagram of QBC based high-gain converter (**b**) Mode-I Operation (**c**) Mode-II Operation.

The high-gain converter shown in Figure 21 that is disclosed in [49], introduces a non-isolated QBC with increase in gain by a factor of three. The QBC uses a VMC in place of the second inductor and a switch capacitor network to increase the gain. A greater voltage gain and less voltage stress on the switch are the outcomes of this change. There is a noticeable improvement over competing non-isolated boost converters thanks to the boost converter’s circuit design. Its shared ground feature and capacity to sustain a steady input current are the sources of this benefit. Our suggested QBC has superior input current and common ground characteristics compared to competing non-isolated high-gain boost converters. Using PLECS software (4.7.6), the study evaluates the outcomes and provides a thorough analysis and design of components. The suggested Quasi-Buck Converter may reach a peak efficiency of about 94% when fed a 48 V input voltage and produces a 385 V output voltage while accounting for component losses. Reducing component-related losses, such as switching and conduction losses, is a viable option for improving efficiency.

(26) $$Gain=\frac{V_{0}}{V_{S}}=\frac{3}{{\left(1-D\right)}^{2}}$$

### 3.4. Summary

The details of the circuit’s topology, their modes of conduction, and the gain equations presented by different possible authors. It demonstrates how to use quadratic boost topology in the operation of a high-gain converter design. In addition to this, numerous research articles have utilized QBC topology to develop innovative high-gain converters. But the design of high-gain converters utilizing the QBC topology necessitates thorough examination of the various issues and constraints that may arise. The achievement and maintenance of high gains is inherently difficult, which requires sophisticated control algorithms and real-time tuning to assure stability. One major restriction that could impact the dependability and lifespan of the system is the voltage stress that components encounter, especially semiconductor devices. Also, many potential uses for these converters could be limited by how sensitive they are to the input voltage range. The complexity of QBC is further compounded by the necessity for accurate output voltage management, electromagnetic interference problems, and efficiency challenges at light load situations. Additional challenges include dealing with transient responses, dealing with non-ideals, guaranteeing safety under different settings, and balancing the economic implications [50,51,52,53,54,55,56]. To overcome these obstacles and maximize the performance of HG-QBC, one must carefully choose components, manage heat, follow safety protocols, and employ exacting design methodologies. High-gain converters provide several kinds of difficulties, which are described in detail in Table 2, along with solutions to these challenges.

## 4. Comparative Analysis

To overcome these challenges, several potential authors have proposed various designs for high-gain converters based on different boosting strategies. Every suggested setup comes with its own set of pros and cons. The design of the converter is influenced by its intended function. Before incorporating high-gain converters in practical scenarios, it is crucial to have a thorough grasp of the different factors linked to these converters. Several high-gain converters using the QBC topology, as suggested by previous researchers, are compared in Table 3. The document presents significant information regarding various converter topologies, including the voltage gain, number of components (S: switch, L: inductor, C: capacitor, D: diode), switching mode, control techniques, input current type, input source, common ground, use cases, efficiency, hardware implementation, cost, and distinguishing features. This comprehensive chart can assist researchers and engineers in making informed assessments in accordance with their specific needs. Furthermore, the comparative analysis illuminates the merits and demerits of every converter topology, enabling a thorough comprehension of their efficacy across various scenarios. By considering each of these elements, engineers and researchers can efficiently select the most appropriate converter topology to meet the requirements of their applications. In addition, the comprehensive chart functions as an asset for forthcoming investigations and advancements in power electronics, establishing a robust groundwork for subsequent progressions in converter technology.

Table 3 (a)–(d) show that there are significant differences in several parameters when comparing different high-gain converter technologies. These include the following: converter type, boosting type, voltage gain, number of components, voltage stress on switches and diodes, control techniques, input current type, input source, usage, efficiency, cost, and specific features. The comparison covers over 26 articles, all of which showcase different converter designs and their characteristics. Several research papers, such as [58,59,60,61,62,63], have been devoted mainly to the search for higher efficiency levels, between 94% and 95.3%, attained by applying sophisticated control techniques and complex circuit modifications. Among these efforts, [58] is noteworthy because it emphasizes the improvement of system stability using soft-switching methods, while [61] focuses on reducing copper losses to improve overall performance. On the other hand, the efficiency metrics reported in [65,80] are comparatively lower, at 90% and 91.4%, respectively. This could be due to different design priorities or implementation challenges. For most contributions, PWM remains the predominant control modality, providing strong regulatory capacities [49,57,64,67,70,76,77]. Different investigations have produced different voltage gain formulas; some have achieved remarkable gains [66,68,72], while other studies have placed more emphasis on the attenuation of stress between components [69,74]. To support a wide range of applications, including sustainable energy, microgrids, electric vehicles, batteries, fuel cells, and renewable energy, all are included in the exploratory scope [49,58,64,70,76]. Notably, studies like [71,75] focus on applications like electric cars and industrial settings, demonstrating a sophisticated strategy catered to industry-specific requirements. A significant number of studies [49,57,58,64,67,76,77] involve hardware realization, but [70,75] give theoretical models without any physical instantiation. Cost factors vary, with certain technologies requiring large implementation investments [58,64,80], while other technologies put cost-effectiveness first [49,76]. Several of the contributions highlight methods for reducing the voltage stress on diodes and switches [58,61,69,74,80], which improve longevity and dependability. Additionally, the widespread use of component optimization methods to minimize stress and ripple [65,72,74] greatly enhances overall performance. These high-gain converter technologies are highly versatile and adaptable due to their tailored features that address specific application demands. Examples of such features include minimizing voltage stresses for microgrid deployments [66,71,75] and reducing output ripple for energy storage systems [76,77].

Researchers have created a wide array of high-gain converter technologies, and this comparison sheds light on them all, revealing their efficiency, performance, and potential domain-specific applications. It stresses the significance of low-cost design, efficient control strategies, and methods for reducing voltage stress in high-gain converters. The research also reveals how people are trying to improve converter performance to meet the changing demands of various power electronics and renewable energy system applications.

## 5. Future Research Directions

Many recent research articles have focused on developing topologies for high-gain converters. These articles pay close attention to critical aspects such as reducing current and voltage stress on power semiconductor devices, implementing soft switching to minimize losses, optimizing structure simplicity, and achieving a high voltage conversion ratio with a low duty ratio. Researchers delve into intricate aspects, conducting comprehensive steady-state analyzes, elucidating operational mechanisms, delineating component design principles, delving into Boundary Conduction Mode, and conducting prototype testing to validate theoretical studies. While derived topologies often focus on steady-state analysis, it is crucial to place a greater emphasis on comprehending the dynamic performance of converters and developing controllers to address real-world circumstances [82,83,84]. After carefully analysing the findings from the mentioned studies, Table 2 highlights the challenges of high-gain converters and suggests potential strategies to address them.

There are several methods to enhance the performance and efficiency of high-gain converters that rely on QBCs. Firstly, it may be feasible to enhance the converter’s efficiency and reduce switching losses by implementing contemporary semiconductor materials and technologies in the switch element. To enhance the converter’s dynamic response and stability, it is possible to implement control algorithm optimizations such as sophisticated modulation techniques or predictive control strategies. Introducing new magnetic components with improved core materials and designs could potentially lead to reductions in size, weight, and losses. Exploring innovative energy storage elements, like supercapacitors or enhanced capacitors, has the potential to enhance our ability to store and transmit energy. Enhancing the dependability and resilience of high-gain converters based on QBC can be achieved through the implementation of adaptive and creative features such as fault-tolerant systems or self-tuning parameters [85,86,87]. The suggested enhancements aim to achieve improved performance, efficiency, and dependability across various converter applications.

**Figure 22:**
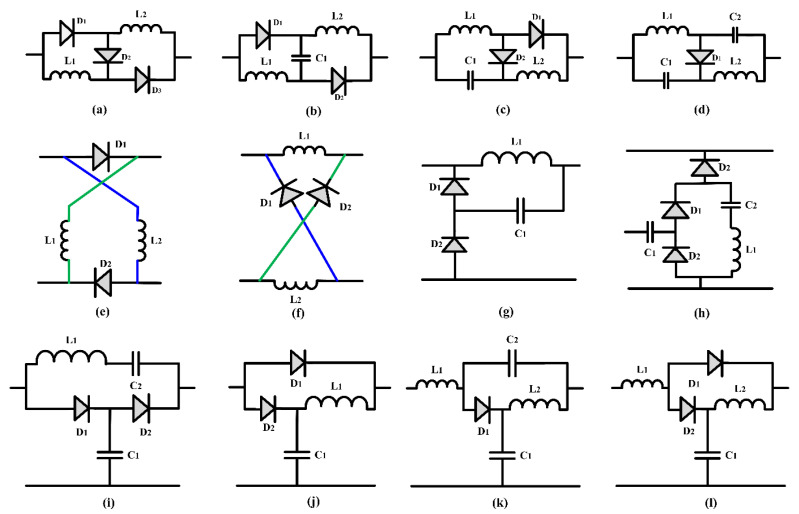
(**a**–**l**) Uncontrolled switched inductor or combination of uncontrolled switch inductor and capacitor based boosting topology.

**Figure 23:**
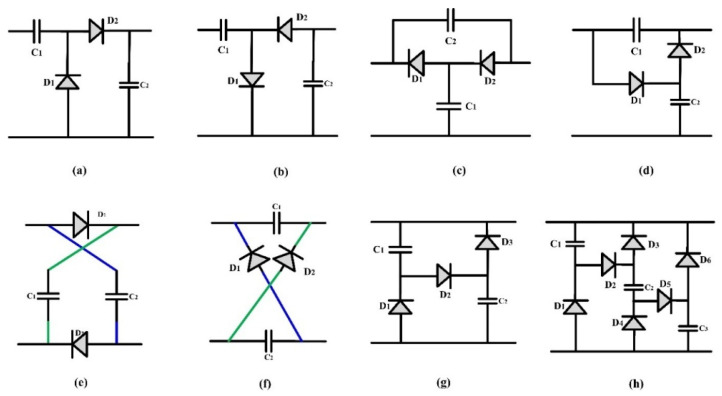
(**a**–**h**) Uncontrolled switch capacitor based boosting topology.

Apart from this, ongoing research also heavily emphasizes the development of new high-gain topologies. Future research efforts will be directed at developing novel high-gain converter topologies that seamlessly integrate conventional converter architectures with distinct boosting techniques. An assortment of various boost techniques based on uncontrolled switch inductors and switch capacitors are illustrated in Figure 22 and Figure 23. By incorporating these advanced technologies into traditional converter topologies, researchers aim to improve the overall performance and efficiency of power conversion systems. The objective of this integration is to develop a noble high-gain converter by merging the best features of traditional converter designs with advanced boosting techniques. Exploring and applying these innovative topologies could potentially revolutionize power conversion, leading to the development of advanced and energy-efficient electrical systems in the future. These advancements promise to significantly enhance energy efficiency, reducing power consumption across various industries and applications. Integrating these cutting-edge topologies into power conversion systems aligns with environmental sustainability goals and offers significant cost savings. Minimizing power consumption and enhancing energy efficiency are crucial in mitigating our environmental footprint and fostering a more sustainable future.

**Future Research Focus:**

❖ Explore design to optimize the performance of HG-QBC, with the goal of achieving higher power density and reducing energy losses.
❖ Enhance switching speeds and minimize conduction losses by utilizing cutting-edge semiconductor materials and techniques, resulting in increased converter efficiency.
❖ Discover adaptive control techniques for HG-QBC in EVs with variable input voltages and loads.
❖ Explore the potential of dynamic algorithms that enhance converter settings in real-time, considering the current operational conditions.
❖ Research on QBC integration with multi-source energy harvesting systems is needed to increase EV energy collection. These systems may use solar, kinetic, or thermal energy.
❖ Make control algorithms that balance and control energy from multiple sources to meet EVs variables power needs.
❖ Verify the robustness and dependability of EVs with HG-QBC, particularly under difficult operating circumstances.
❖ Develop innovative cooling and packaging methods to strengthen the converter’s resistance to vibrations, temperature changes, and other harsh environments.
❖ Focus investigation on HG-QBC bidirectional power flow to make ability of EVs can use for V2G and G2V connections.
❖ Develop coordinated control methods to simplify smart grid integration so EVs can stabilize and support demand response programs.
❖ Identify possible areas for cost reduction and assess the economic viability of EVs with HG-QBC.
❖ Investigate new production methods, affordable supplies, and scalable designs to lower converter prices for EVs markets.
❖ Contribute to the establishment of industry standards for HG-QBC base EVs to ensure seamless interoperability and compatibility across various EVs and charging infrastructures.
❖ Collaborate with appropriate regulatory agencies and business organizations to create universally applicable testing protocols and specifications.

## 6. Conclusions

This review article has explored the crucial role of DC-DC converters in EV applications, specifically highlighting the transition to high-gain boost converters. To optimize performance, it is essential to utilize a high-gain converter based on a QBC. The importance of DC-DC converters in EVs and the rationale for using high-gain boost converters are elucidated in an informative way. Conventional QBC circuit design and its analysis has been confirmed through experimental study. Furthermore, the article provided an exhaustive overview of the characteristics and designs of high-gain converters based on the QBC topology and an analytical evaluation of several approaches utilized for their design. Moreover, the survey highlighted the limitations and downsides of the present-day high-gain converters, such as their high cost and poor efficiency. It also highlighted the importance of fixing these issues so high-gain converters can be used to their maximum potential in EVs. The results of this survey will guide future studies and innovations in high-gain converter technology, which will benefit the EV industry.

## Figures and Tables

**Table 1:** Comparative summary of different boost converter topologies.

Feature	Quadratic Boost Converter	Interleaved Boost Converters	Cascade Boost Converters	Conventional Boost Converters
Operating Principle	The high step-up ratio is achieved by using quadratic terms in the inductor current.	Reducing input/output current ripple is achieved through the interleaving of many channels.	Increase the voltage conversion ratio by stacking converters.	Step-up of voltage via a single power stage.
Voltage Gain	Very high	High	Very high	Moderate to high
Ripple Current	Low to Moderate	Low	Low to Moderate	Moderate to High
Size and Weight	Moderate	Moderate to Large	Large	Small to Moderate
Components Count	Moderate	High	High	Low to Moderate
Complexity	Moderate	High	High	Low to Moderate
Applications	High step-up voltage applications, LED drivers, renewable energy systems, electric vehicles	High power applications, PV systems, electric vehicles	Renewable energy systems, grid-tied inverters	General voltage boosting applications
Recent Technological Developments	Design and control	Advances in control strategies	Cascade control techniques	Continuous improvements

**Table 2:** Challenges associated with high-gain converter and their mitigation techniques.

Challenges/Limitations	Description	Mitigation Strategies
Efficiency	Increasing the voltage gains leads to an increase in the switching losses.	Improve efficiency by incorporating advanced switching topologies.
Output Voltage Ripple	The ripple in the output voltage has increased.	Utilize advanced filtering methods, such as LC filters, to reduce ripple.
Complex Control Algorithms	Advanced control algorithms and real-time adjustments are necessary for achieving high voltage gains while maintaining stability.	Enhance the flexibility and adaptability of your system by utilizing digital controllers and sophisticated control algorithms.
Voltage Stress on Components	The high voltage levels put stress on diodes and transistors, leading to decreased efficiency and dependability.	Carefully consider technologies that can handle greater voltages and make sure to select components with precision.
Input Voltage Range Sensitivity	Variations in input voltage may limit the system’s applicability, requiring the addition of extra circuitry or control methods to guarantee proper functioning.	Use voltage regulation techniques and construct circuits for input voltage conditioning.
Efficiency at Light Load Conditions	The inefficient light loads can be hampering the overall energy efficiency of the system.	Improve circuit design to increase efficiency across various load circumstances and utilize low-power modes.
Reliability	The Reliability can decline as component stress increases.	Perform thorough reliability testing and set up backup systems for crucial applications.
Electromagnetic Interference (EMI)	A high switching frequency causes EMI, requiring EMI filters and EMC compliance.	To reduce electromagnetic interference, make sure that shielding, filtering, and compliance with EMC standards are implemented properly.
Precise Output Voltage Regulation	Meticulous design considerations are necessary for precise output voltage regulation in dynamic circumstances.	Use specific components, include regulation circuits, and set up feedback control systems to guarantee precise performance.
Transient Responses	Performance may be impacted by controlling transient responses during load or input changes and parasitic elements.	Optimize circuit design for maximum performance by carrying out comprehensive evaluations and implementing compensating plans.
Size and Weight	It may be necessary to use larger inductor and capacitor sizes to meet the system’s requirements.	Discover cutting-edge component technologies and lightweight materials to enhance performance.
Complexity	Complex configurations of the control and feedback systems have developed to fulfill the requirements of sophisticated applications.	Work with experts in control systems to simplify processes and create cutting-edge automation algorithms.
Cost Implications	The components and design complexity may raise production costs, affecting the system’s economic feasibility.	Analyze affordable options that satisfy exacting performance standards.
Temperature Management	Increased system temperatures are caused by amplified power losses.	To maximize cooling efficiency, develop and implement cutting-edge thermal management technologies.
Scalability	Scaling at different power levels presents challenges.	Create systems that can easily adjust to changing requirements by collaborating with experts in power systems.
Practical Implementation Challenges	Integrating the system with existing systems poses certain challenges.	To enable smooth integration and carry out comprehensive field testing, work closely with industry partners.
Application Range	Extensive usage of the technology in low-power applications is not feasible.	Collaborating with domain experts to provide individualized solutions, investigate hybrid solutions to increase application scope.

**Table 3:** **(a)–(d)** Comparing various high-gain converter technologies developed by different researchers.

**(a)**																					
References					[49]		[57]			[58]		[59]	[60]	[61]			[62]		[63]		
Converter type					M-QBC-1		Bidirectional QBC			QBC-1		M-QBC-2	M-QBC-3	QBC-2			M-QBC-4		M-QBC-5		
Boosting type					VMC		QBC			QBC		Switching Capacitor	coupled inductor	QBC			Switch Inductor		Switch Inductor and Capacitor		
Voltage gain					$\frac{3}{{\left(1-D\right)}^{2}}$		$\frac{1}{{\left(1-D\right)}^{2}}$			$\frac{1}{{\left(1-D\right)}^{2}}$		$\frac{3}{{\left(1-D\right)}^{2}}$	$\frac{3-D}{{\left(1-D\right)}^{2}}$	$\frac{1}{{\left(1-D\right)}^{2}}$			$\frac{1}{{\left(1-D\right)}^{2}}$		$\frac{1}{{\left(1-D\right)}^{2}}$		
No of components				S	1		4			1		2	1	1			2		1		
				L	3		2			2		2	3	2			2		2		
				C	4		2			2		4	3	2			2		2		
				D	7		0			3		4	5	3			2		3		
Voltage stress (Switch)					$\frac{2V_{0}}{3}$		$V_{0}(2-D)$			$\frac{V_{0}}{2}$		$\frac{V_{s}}{{\left(1-D\right)}^{2}}$	$\frac{3V_{s}}{\left(1-D\right)}$	$\frac{V_{0}}{2}$			$\frac{V_{s}}{{\left(1-D\right)}^{2}}$		$\frac{V_{s}}{{\left(1-D\right)}^{2}}$		
Voltage Stress (Diode)					$\frac{2V_{s}}{\left(1-D\right)}$		$\frac{V_{s}}{\left(1-D\right)}$			$V_{0}$		$\frac{2V_{s}}{{\left(1-D\right)}^{2}}$	$\frac{2V_{s}}{{\left(1-D\right)}^{2}}$	$V_{0}$			$\frac{(2-D)V_{s}}{{\left(1-D\right)}^{2}}$		$\frac{2V_{s}}{{\left(1-D\right)}^{2}}$		
Control Techniques					PWM		PWM			SMC		Soft switching/PWM	PWM	current-control			average current mode control		PWM		
Input current type					Continuous		Non pulsating			Pulsating		continuous	Continuous	Pulsating			Non pulsating		Continues		
Input Source					Renewable		battery			PV		Solar	DC Source	DC Source			Solar/FC		Solar		
Common ground					Yes		Yes			Yes		Yes	No	Yes			Yes		No		
application					Sustainable Energy		EV/HEV			Microgrid		sustainable energy	Microgrid	sustainable energy			EV		Renewable energy		
efficiency					94%		94.7%			95%		95.3%	94.8%	94%			95%		94.4%		
Hardware Implementation					Yes		Yes			Yes		Yes	Yes	No			No		No		
Cost					Minimum		High			Low		High	High	Low			Medium		Medium		
Feature					Reduces the switch’s voltage stress		Absolute common ground with a broad voltage spectrum			System design more stable.		ZVS and ZCS reduce switching losses and reverse recovery.	High voltage strains are reduced via passive clamping circuits.	Enhanced performance and stability			Reducing the copper losses		Reduced capacitor voltage stress		
**(b)**																					
References					[64]		[65]			[66]			[67]				[68]		[69]		
Converter type					QBC-3		M-QBC-6			M-QBC-7			M-QBC-8				Quadratic Buck-Boost		M-QBC-9		
Boosting type					Interleaved		Switched Capacitor			Switched Inductor			Coupled Inductors				Quadratic		Switched Inductor		
Voltage gain					$\frac{4}{{\left(1-D\right)}^{2}}$		$\frac{3-D}{{\left(1-D\right)}^{2}}$			$\frac{3+D+n(1-D)}{{\left(1-D\right)}^{2}}$			$\frac{\left(n^{2}+(1+n)(3+D)\right)}{{\left(1-D\right)}^{2}}$				${\left(\frac{D}{\left(1-D\right)}\right)}^{2}$		$\frac{4-4D+D^{2}}{{\left(1-D\right)}^{2}}$		
No of components				S	4		2			2			2				2		2		
				L	5		2			4			2				3		2		
				C	5		4			5			5				3		4		
				D	7		4			5			5				2		4		
Voltage stress (Switch)					$\frac{V_{0}}{4}$		$\frac{(2-D)V_{0}}{{\left(1-D\right)}^{2}}$			$\frac{(1-D)V_{0}}{3+D+n(1-D)}$			$\frac{(1+n+nD)V_{0}}{\left(n^{2}+(1+n)(3+D)\right)}$				$\frac{1-D}{D^{2}}$		$\frac{V_{s}}{{\left(1-D\right)}^{2}}$		
Voltage stress (Diode)					$\frac{V_{0}}{2}$		$\frac{2V_{0}}{{\left(1-D\right)}^{2}}$			$\frac{(2+n(1-D))V_{0}}{3+D+n(1-D)}$			$\frac{(n^{2}+2+3n)V_{0}}{\left(n^{2}+(1+n)(3+D)\right)}$				$\frac{1-D}{D^{2}}$		$\frac{V_{s}}{{\left(1-D\right)}^{2}}$		
Control techniques					PWM		PWM			Voltage Control			PWM				PWM		Voltage Control		
Input Source					Renewable Energy		Renewable Energy			Solar			Solar				Renewable Energy		PV		
Common ground					No		Yes			Yes			Yes				Yes		Yes		
Application					microgrid		Microgrid			Microgrid			Microgrid				Grid		Energy Storage		
Efficiency					95.82%		90%			96.28%			94.3%				95.96		93.6		
Hardware implementation					Yes		Yes			Yes			Yes				Yes		Yes		
Cost					V. High		High			V. High			V. High				Medium		High		
Feature					Reduce the Current Ripple		Low voltage stresses			Less stress on the voltage across the output diodes			Switches and diodes have minimal voltage stress.				Decreased input/output capacitor current stress		Low stress across devices		
**(c)**																					
References					[70]			[71]			[72]		[73]		[74]			[75]			
Converter type					Quadratic Buck-Boost			Quadratic Boost–Cuk			QBC-4		QBC-5		Quadratic Buck-Boost			M-QBC-10			
Boosting type					Quadratic			Quadratic			Quadratic		Quadratic		Switch Capacitor			Voltage Doubler			
Voltage gain					${\left(\frac{D}{\left(1-D\right)}\right)}^{2}$			$\frac{1+D}{{\left(1-D\right)}^{2}}$			$\frac{1}{{\left(1-D\right)}^{2}}$		$\frac{1}{{\left(1-D\right)}^{2}}$		$\frac{D}{{\left(1-D\right)}^{2}}$			$\frac{2n+1+D}{{\left(1-D\right)}^{2}}$			
No of components				S	1			1			1		2		2			2			
				L	3			3			2		2		2			2			
				C	3			4			2		3		2			5			
				D	5			4			3		3		2			5			
Voltage stress (Switch)					$\frac{V_{s}}{D^{2}}$			$\frac{D(2+D+D^{2})V_{0}}{{\left(1-D\right)}^{2}}$			$\frac{V_{0}}{2}$		$\frac{V_{0}}{2}$		$\frac{V_{0}}{2}$			$\frac{(1+D)V_{0}}{\left(2n+1+D\right)}$			
Voltage stress (Diode)					$\frac{(1-D)V_{s}}{D^{2}}$			$\frac{D(1+D)V_{0}}{{\left(1-D\right)}^{2}}$			$V_{0}$		$V_{0}$		$V_{0}$			$\frac{(1-D)V_{0}}{\left(2n+1+D\right)}$			
Control techniques					Voltage Control			Voltage Control			Sliding-Mode Controllers		Modified Sliding-Mode Controllers		Voltage Control			Voltage Control			
Input Source					Battery			Fuel Cell			DC Source		Solar		Battery			Renewable Energy			
Common ground					Yes			Yes			Yes		Yes		Yes			Yes			
Application					Industrial applications			EVs			EVs		Microgrid		Industry			Microgrid			
Efficiency					92%			94%			94.4%		89%		91.4%			94.3%			
Hardware implementation					No			Yes			No		Yes		Yes			No			
Cost					High			Medium			Low		Low		Medium			High			
Feature					Simple construction design			Low voltage stress on switch and output components			A fixed-frequency PWM-based SM controller is proposed.		discusses DCM operates at variable switching frequency.		Zero output voltage ripple is possible.			Low input/output current ripple			
**(d)**																					
References					[76]			[77]			[78]		[79]		[80]			[81]			
Converter type					M-QBC-11			QBC-6			Ultrahigh Boost		M-QBC-12		Buck-Boost			M-QBC-13			
Boosting type					Switched Capacitor			QBC			Voltage multiplier		Multiplier Cell		Zeta			Coupled Inductor			
Voltage gain					$\frac{1}{{\left(1-D\right)}^{2}}$			$\frac{1}{{\left(1-D\right)}^{2}}$			$\frac{5-D(4-D)}{{\left(1-D\right)}^{2}}$		$\frac{1+D}{{\left(1-D\right)}^{3}}$		$\frac{D^{2}}{{\left(1-D\right)}^{2}}$			$\frac{3+2D}{{\left(1-D\right)}^{2}}$			
No of components				S	2			2			2		2		1			1			
				L	2			3			2		3		3			4			
				C	2			5			4		3		4			4			
				D	2			5			4		2		4			5			
Voltage stress (Switch)					$\frac{V_{0}}{2}$			$\frac{V_{0}}{2}$			$\frac{(1-D)V_{0}}{5-D(4-D)}$		$\frac{2V_{s}}{{\left(1-D\right)}^{2}}$		$\frac{D^{2}V_{0}}{{\left(1-D\right)}^{2}}$			$\frac{V_{0}}{3+2D}$			
Voltage stress (Diode)					$\frac{V_{0}}{2}$			$V_{0}$			$\frac{(2-D)V_{0}}{5-D(4-D)}$		$\frac{(1-D)V_{s}}{{\left(1-D\right)}^{2}}$		$\frac{V_{0}}{{\left(1-D\right)}^{2}}$			$\frac{{(2+D)V}_{0}}{3+2D}$			
Control Techniques					PWM			Modified Current			Voltage Control		Modified Voltage		Voltage Control			PWM			
Input Source					Renewable energy			DC Source			Battery		Renewable energy		DC Source			Fuel Cell			
Common ground					Yes			Yes			Yes		Yes		Yes			No			
Application					Low Energy Storage			Microgrid			EVs		Microgrid		Renewable energy			EVs			
Efficiency					94.5%			93.4%			96.4%		93.6		94.2%			94.5%			
Hardware implementation					Yes			No			Yes		No		Yes			Yes			
Cost					Medium			Low			High		High		Medium			V. High			
Feature					Minimize the output voltage ripple			Propose voltage-mode control techniques			higher gain with high efficiency.		Ultra-high level of voltage gains with a low stress across diode		Low voltage stress on diode			Voltage stress on the power switch is less			

## Data Availability

The data used in the current study can be accessed from the corresponding authors depending on the purpose of use.
